# Tailored and Integrated Web-Based Tools for Improving Psychosocial Outcomes of Cancer Patients: The DoTTI Development Framework

**DOI:** 10.2196/jmir.2849

**Published:** 2014-03-14

**Authors:** Rochelle Smits, Jamie Bryant, Rob Sanson-Fisher, Flora Tzelepis, Frans Henskens, Christine Paul, William Stevenson

**Affiliations:** ^1^Priority Research Centre for Health BehaviourUniversity of Newcastle & Hunter Medical Research InstituteCallaghanAustralia; ^2^School of Electrical Engineering and Computer ScienceFaculty of Engineering and Built EnvironmentUniversity of NewcastleCallaghanAustralia; ^3^Department of HaematologyRoyal North Shore HospitalUniversity of SydneySt LeonardsAustralia

**Keywords:** Internet, consumer health information, health literacy, medical informatics, neoplasms, communication

## Abstract

**Background:**

Effective communication with cancer patients and their families about their disease, treatment options, and possible outcomes may improve psychosocial outcomes. However, traditional approaches to providing information to patients, including verbal information and written booklets, have a number of shortcomings centered on their limited ability to meet patient preferences and literacy levels. New-generation Web-based technologies offer an innovative and pragmatic solution for overcoming these limitations by providing a platform for interactive information seeking, information sharing, and user-centered tailoring.

**Objective:**

The primary goal of this paper is to discuss the advantages of comprehensive and iterative Web-based technologies for health information provision and propose a four-phase framework for the development of Web-based information tools.

**Methods:**

The proposed framework draws on our experience of constructing a Web-based information tool for hematological cancer patients and their families. The framework is based on principles for the development and evaluation of complex interventions and draws on the Agile methodology of software programming that emphasizes collaboration and iteration throughout the development process.

**Results:**

The DoTTI framework provides a model for a comprehensive and iterative approach to the development of Web-based informational tools for patients. The process involves 4 phases of development: (1) Design and development, (2) Testing early iterations, (3) Testing for effectiveness, and (4) Integration and implementation. At each step, stakeholders (including researchers, clinicians, consumers, and programmers) are engaged in consultations to review progress, provide feedback on versions of the Web-based tool, and based on feedback, determine the appropriate next steps in development.

**Conclusions:**

This 4-phase framework is evidence-informed and consumer-centered and could be applied widely to develop Web-based programs for a diverse range of diseases.

## Introduction

### Global Burden of Cancer and its Psychosocial Consequences

Cancer is one of the leading causes of death world-wide, accounting for 7.6 million deaths in 2008 [1]. A diagnosis of cancer can impose a significant psychological burden on patients and their families. Challenges include coping with uncertainty surrounding prognosis, making important treatment decisions, and learning how to manage often debilitating physical, psychological, and social effects of the disease. Cancer care is complex, involving a multidisciplinary team, including general practitioners, cancer doctors, nurses, and other allied health professionals, and patients often have to travel for treatment [2]. Between 32% and 48% of individuals diagnosed with cancer experience psychological distress, including anxiety and depression [3]. Failure to address these issues through the provision of appropriate information and psychosocial care may have a significant impact on clinical patient outcomes and the health care system, including higher frequency and intensity of physical symptoms [4], poorer adherence to treatment regimes [5], and increased utilization of medical services [6].

### The Importance of Effective Communication in Reducing Psychosocial Burden

Effective communication and provision of information to cancer patients and their families about their disease, treatment options, and possible outcomes improve psychosocial outcomes [7,8]. Recognition of the importance of effective communication has been driven by increased consumer activism, as well as legal imperatives to ensure patients are well informed of their treatment options and are able to exercise control over their role in making decisions regarding their care [9]. In order to make an informed decision, for example, a patient must be provided with clear and sufficient information about the risks and benefits of available treatment options [10]. Failing to fully inform patients about their condition, treatment options, and potential consequences, as well as misrepresenting information, can lead to legal challenges and medical litigation [11]. This has led to a shift from paternalistic approaches to information provision and disclosure within the health care system to a model that emphasizes autonomy and patient-centered care [12], which are reflected in changes to legislation, bioethical guidelines, and accepted principles within the medical profession [12-15].

The philosophy of patient-centered care promotes self-management and patient empowerment by emphasizing patients as partners in decision making and offering tailored health care that is responsive to patient needs [16]. Effective patient-centered care is associated with improved health outcomes, enhanced patient and practitioner satisfaction, and decreased use of health care services [16]. The Institute of Medicine report, “Crossing the Quality Chasm”, promoted patient-centered care as an essential component of quality health care [12], a position that is passionately supported by consumer advocates. The involvement of consumers in health service reform and research is essential to ensuring patients’ needs, values, and preferences are represented [17]. The health consumer movement has strengthened in recent decades with the establishment of consumer advocacy groups, including MacMillan (United Kingdom) [18], Cancer Voices (Australia) [19], and LiveStrong (United States) [20]. Consumers are actively involved in campaigns to engender change in health care policy and practice, creating awareness of diseases and offering support to patients, and determining research priorities and distribution of funding [21]. The sense of autonomy and assertiveness promoted by the health consumer movement has led to an appeal by patients for active involvement in decision making in partnership with their health care provider [22].

### Accounting for Individual Patient Variation in Communication

Ensuring patients are well informed and able to participate in making complex decisions about their care is complicated by several factors. As well as providing patients with information, there is a need to ensure that information is provided in a way that is understood and recalled. Health literacy refers to an individual’s ability to obtain, process, and understand health information to make appropriate decisions about their health care [23]. Around one in five adults in the United States has low health literacy [23]. Individuals with low health literacy often face challenges in acting as fully informed consumers. Consequently, they may lack a clear understanding about their condition, their options for and the potential consequences of treatment [24], and therefore find it difficult to be active in making decisions about their health care [23]. Given the increasingly complex treatment options available to cancer patients, health literacy must be an important consideration when providing information to patients [23].

### Accounting for Clinician Variation and Patient Preferences in Communication

Patients often report that clinicians do not provide information about their diagnosis and treatment options in ways they can understand [23]. This mismatch between the information provided by clinicians and the patient’s level of health literacy hinders the ability of patients to recall and utilize the information [23]. There is also significant variation in patient preferences for involvement in medical decision making [25]. Degner and colleagues found that 22% of breast cancer patients preferred to make the decision about their treatment, 44% wanted a collaborative approach between themselves and their clinician, and 34% of women preferred their clinician having responsibility for treatment decisions [25]. Achieving the desired level of involvement in decision making may be further hindered by the ability of clinicians to accurately assess patient preferences. Several studies have revealed that clinician perceptions are incongruent with cancer patient preferences for involvement in medical decision making in approximately 58% of cases [25,26].

## The Limitations of Traditional Approaches to Information Provision

There are a number of existing approaches to providing information to patients. These include patient-clinician interaction, written/printed leaflets and booklets, audio-visual materials, and more recently, websites accessed via the Internet. However, these approaches have a number of limitations. For example, patients consistently report being dissatisfied with the amount and quality of the information they receive directly from clinicians [27]. Health care professionals may not be aware of patients’ needs and preferences for receiving information [26], and clinicians are known to have poor accuracy in tailoring information to match patient preferences [8,26]. Inadequate communication or withholding information by the health care provider can lead to poor recall or misunderstanding of information [27].

In addition, written information materials, including leaflets and booklets, often have a reading level that is higher than that of the majority of the population [8,28]. This discrepancy results in information materials that are not readily accessible to some consumers and have the potential to cause unnecessary confusion and anxiety. The most vulnerable consumers—those with low literacy and numeracy—are particularly likely to be affected by the challenges of written information. Varying learning styles and literacy levels may also make it difficult for patients to absorb and engage with written information [29]. Written information materials also depend on the consumer having adequate visual capabilities and therefore are not suitable for visually impaired groups. Currency of information is also important given ongoing developments in the health research literature. Regularly updating the content of printed materials to reflect current best evidence is often not cost-effective, resulting in out-of-date materials remaining in circulation [30]. Written and audio-visual information including videos, DVDs, and CD-ROMs also cannot be tailored to the individual needs of patients and are often very expensive to produce and update as new evidence becomes available [30].

## The Unrealized Potential of Web-Based Information

Heralded as the future for providing patients with health information in the mid-1990s [30-32], the amount of health information on the Internet has grown exponentially. Surveys of cancer patients and their families have shown that 62-80% have an interest in obtaining information and support via the Internet [33,34]. Patients also frequently report the Internet as an important source of information about diagnosis and treatment [35,36]. The Internet shows great promise in reaching a large proportion of the community; 78% of citizens in the United States have Internet access [37], and new technologies including smartphones and tablet computers have led to a growth in accessing the Internet via mobile devices [38]. Mobile technology improves convenience for users and allows people to connect with Web-based services and information anywhere at any time, which is a feature reported by cancer patients as the main benefit of Web-based information [34]. This provides an ideal platform on which to develop information interventions for improving patient outcomes and health care delivery to a wide audience.

However, current Web-based information is not without limitations. While the Internet plays an important role in meeting the informational needs of many patients [35], the unmonitored provision of information, coupled with unstructured and unassisted use, has the potential to negatively impact psychological well-being and patient outcomes. There are growing concerns, for example, about the quality of health information available on the Internet [35,39]. Information is often provided without any regulation of credibility of authorship or accuracy of content. This has the potential for dangerous and negative consequences, particularly given that around half of all people who search for health information on the Internet do not discuss the information with their health care provider [40]. The reading level required for many cancer information websites is also far above that of the average population. Friedman and colleagues report that 64% of cancer websites are written at a level of grade 13 or higher [41], which suggests that a large proportion of information may not be accessible by less literate patients. Lack of specificity, complexity, and being too impersonal are also perceived as disadvantages by patients [34].

These findings highlight the need for more accurate, evidence-based information that is easily understood by consumers, developed in close co-operation with health care professionals, and integrated into clinical care in order to overcome the dangers of misinformation. There is an urgent need to develop a sustainable, systematic, and integrated approach to providing information in a way that addresses health literacy, increases patient involvement in decision making and their care, and operates independently of resource constraints and other barriers that deter the routine delivery of tailored information.

## New-Generation, Integrated, and Tailored Web-Delivered Tools as a Solution for Effective Information Provision

### Overview

In contrast to the passive dissemination of information via traditional Web-based approaches, new-generation Web-based technology offers an innovative and pragmatic solution to the shortcomings of general Web-based information by providing a platform for interactive information seeking, information sharing, and user-centered design.

### Self-Tailored Content

Individual patient preferences for information vary. Some patients wish to obtain as much information as possible (monitors), while others prefer to avoid potentially threatening information (blunters) [42]. Tailoring information to match individual preferences improves psychosocial outcomes [12,43] by preventing unnecessary anxiety and increasing recall of information [44]. Web-based information tools have the potential to empower patients to determine when and how often they access information and the type and amount of information they would like, allowing them to become actively involved in their health care. This improves on traditional clinician-delivered information provision where patient preferences may not be considered.

There is also significant potential for Web-based tools to modify content to account for individual factors such as health literacy and cultural appropriateness. For example, upon login, the user may be asked to complete a brief screening questionnaire, indicating information such as their ethnicity, highest level of education, and preference for detailed versus brief descriptions. These data can be used to tailor the Web-tool content to suit the needs and preferences of the user, for example, by increasing the number of pictures and videos presented or including culturally relevant vocabulary and images. In addition, options can be made available to customize the language (eg, English, Japanese, Spanish) in which the information is presented or increase the size of the text to assist the visually impaired. The interactive nature of Web-based tools allows for real-time customization that is not offered by printed information materials.

### Clinician-Tailored Content

Algorithms can be used to select the appropriate information to be presented in Web-based information tools based on data entered by the patient or their clinician. However, clinicians should also play a role in tailoring the information presented within a Web-based tool to ensure it is suited to the user’s unique medical circumstances. For example, a Web-based tool can tailor the information presented based on data entered by the clinician regarding the patients’ diagnosis and relevant treatment options. Customizing the content in this way may prevent the patient from feeling unnecessary distress or false hope over viewing treatment information that is not appropriate for their circumstances.

The involvement of clinicians in tailoring content is important for preventing potential harm caused by misinformation and ensuring the information is relevant to individual patients and their families. Cancer diagnoses can be very complex, and the resulting discussions about treatment options, side effects, and prognosis may require patients to absorb a large amount of complicated information within a short timeframe. The provision of a Web-based information tool that is tailored by the clinician to the unique circumstances of the individual can act as a reliable source of information, supplementary to the doctor-patient consultation. Web-based tools tailored in this way have the capacity to overcome issues such as misinformation [35,37-39], patient comprehension, and information recall [45].

### Multiformat Presentation of Information

The ability to understand and recall medical information is important for ensuring adherence to recommendations for care [45]. Studies have reported that 40-80% of medical information is forgotten, and about half of the information provided to patients is remembered incorrectly [45]. Levels of anxiety can also negatively impact patient recall of information [45], which is particularly relevant for patients facing a life-threatening illness. A combination of written, spoken, and visual formats has been recommended for improving recall of information [45]. Web-based information tools have the potential to deal with issues surrounding patient memory for medical information and allow information to be presented in multiple formats (eg, diagrams, videos, text) to suit different learning styles [30] and literacy levels [45].

### Connecting People and Information Sharing

New generation Web technology provides an effective way of connecting patients and families with information and support regardless of their location. For example, social networking sites, online discussion forums, and video-conferencing allow patients and carers, from various and remote locations, to be part of a virtual community, share their experiences and offer support to one another. The availability of this additional support network, which may be otherwise inaccessible, has the potential to improve socialization and reduce feelings of isolation often experienced by patients and their families dealing with a life-threatening illness such as cancer [46]. A survey of hematological cancer survivors found that the second highest ranked item of high-level unmet need was “finding someone to talk to who understands and has been through a similar experience” [47].

The availability of Web technology encourages the involvement of family and friends in information seeking. Family and friends are able to access information they may wish to know but are reluctant to seek from health care professionals for fear of upsetting the patient, such as information about prognosis, survival, or long-term complications. A Web-based information tool provides a discrete mechanism for accessing this information without the issues of information quality and accuracy often associated with Internet resources. This may allow loved ones to better plan for the future. The instantaneous nature of the Internet also allows for information to be easily shared between patients, their families, and friends. Users are able to access the same information at the same time, send information and links via email, share information and links using social networking sites, and discuss the information using video-conferencing software, such as Skype.

While Web-based tools allow for information to be shared quickly and easily, there are potential privacy issues that must be considered. In the United States, the Privacy Rule issued under the Health Insurance Portability and Accountability Act of 1996, governs the way in which health information can be shared in order to adequately protect the privacy of individuals while allowing the information flow necessary for the provision of high quality health care [48]. Similarly, a number of countries have legislation covering the recording, storage, and transmission of personal health information. Where Web-based tools require individuals’ health information to be provided by their clinician and stored on a secure server in order to allow for sufficient tailoring of information, it is critical to design the system with a clear understanding of implications of the relevant legislation and in a manner that ensures that adequate privacy and data protections are in place to prevent unauthorized access or sharing of private health information. In addition, where Web-based tools are used to share information with friends and family as described above, it is essential that the tool allows for the patient, or an authorized proxy, to have control of what information is made available to others.

### Inclusion of Decision Support Tools

The International Patient Decision Aids Standards (IPDAS) Collaboration [49] describes decision aids as evidence-based tools designed to prepare a person for making a decision about their health care options through deliberative exercises such as value weighting [50,51]. Web-based information tools offer a unique opportunity to seamlessly integrate decision aids with the provision of health care information. Web-based tools allow decision aids to be interactive and presented in multiple formats incorporating video, images, and animations, all of which are useful for presenting risk information to patients.

### Integration With Clinical Care Rather Than Passive Patient-Directed Information Seeking

Web-based information tools may facilitate communication with health care providers. The use of a question prompt sheet within consultations has been shown to be effective in increasing patient participation and reducing unmet information needs [52]. The interactive nature of Web-based tools may enhance the generation and utilization of prompt sheets for patients by tailoring content to correspond with the areas of most interest to the patient. For example, a Web-based information tool has the capacity to generate a question prompt sheet based on the topics of information accessed by the user. The Internet also offers the additional advantage of allowing prompt sheets to be automatically emailed to the health care provider prior to the consultation. This may improve the relevance of the information provided to the patient within the consultation due to increased preparation by the health care provider.

### Distribution, Maintenance, and Feedback

The ability to track and record how the website is used provides an accurate and detailed process measure for evaluating the intervention. Delivery and maintenance of Web-based information tools have the potential to be highly cost-effective [53] and can be easily updated in a timely manner to reflect current best evidence.

## Framework for Developing an Integrated Web-Based Information Tool

### Overview

Several reviews of the literature have examined the effectiveness of Web-based interventions for patient education, psychosocial care, and support [54-57]. While these reviews indicate some benefit of Web-based interventions on patient outcomes including knowledge, social support, health behaviors, and psychosocial well-being, findings are mixed and conclusions limited by the methodological weaknesses of the studies examined. Heterogeneity in the methods used to produce and deliver these interventions may contribute to inconclusive findings. There is often a lack of clear, explicit descriptions of the procedures for developing Web-based interventions in the literature, impeding replication and translation of effective interventions. While there are many useful resources available to guide the development of patient information materials in general [58-60], there is a need for a systematic process for the development of patient education and support interventions delivered specifically via the Internet.

We propose a framework for developing Web-based information tools that draws on our experience of constructing such a tool for hematological cancer patients and their families. The framework is based on principles proposed for the development and evaluation of complex interventions [61,62], which emphasize the importance of using a phased approach, starting with needs assessment, pilot work, and moving on to an exploratory and then definitive evaluation. The framework draws on the Agile methodology of software programming [63], which emphasizes collaboration and iteration throughout the development process. The Agile methodology allows for projects to evolve and be responsive to change, as programmers, researchers, and stakeholders are able to interact to shape the direction of the project through all phases of development.

**Figure 1:**
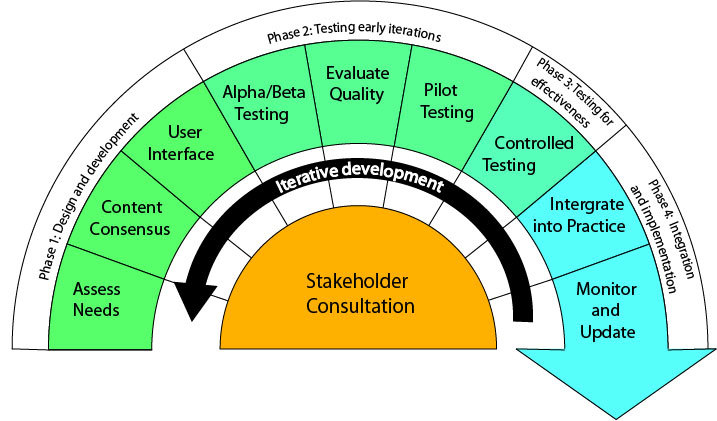
The DoTTI framework for the iterative and consultative development of a Web-based information tool.

Figure 1 illustrates the DoTTI framework for developing a Web-based information tool. The process involves four phases of development: (1) design and development, (2) testing early iterations, (3) testing for effectiveness, and (4) integration and implementation. At each step, stakeholders (including researchers, clinicians, consumers, and programmers) are engaged in consultations to review progress, provide feedback on versions of the Web-based tool, and based on feedback, determine the appropriate next steps in development. Stakeholder participation and iteration have been identified as being essential to the development of effective eHealth technologies [64]. The phases of development are not intended to represent what could be seen as a linear or waterfall approach [65] to the creation of the software underpinning the tool. Rather, the phases represent a staged and concurrently iterative process to the creation of the tool, noting that the development of the informational content and the implementing technology are entwined.

### Phase 1: Design and Development

#### Identify the Target Population and Conduct a Needs Assessment to Determine Patients’ Information and Support Needs

Understanding the needs of the target population is the crucial first step for delivering high quality, patient-centered care [66]. Many existing needs assessment tools assess cancer patients’ needs across a range of domains including physical, psychological, social, financial, and information [66]. While information needs vary, up to 97% of cancer patients report unmet information needs [67]. Common areas where cancer patients report needing additional information include being informed about self-care strategies and the benefits and side effects of treatment [67]. Furthermore, cancer patients tend to report greater unmet needs during the treatment phase [67]. Prior to developing a Web-based information tool, a needs assessment that identifies the informational and support needs of the target population should be undertaken.

#### Build on Existing Information Resources and Gain Consensus From Health Care Providers

Building a partnership with key patient support organizations within the field of interest is advantageous for sharing of resources and expertise. Many support organizations have an established set of information resources available for the target population, which may be used with permission to form the foundation of the Web program content.

Involving health care professionals in the production, implementation, dissemination, and evaluation of Web-based information tools may increase patients’ use of the tool, adoption by health care organizations, and effectiveness and acceptability [39]. At least one advisory group should be established to provide guidance, advice, and feedback on the content of the intervention and to obtain consensus regarding this information. Expert advisory groups should comprise multidisciplinary health care providers as well as members of key support organizations, so that a wide range of views can be incorporated into the intervention. Building cooperative and respectful relationships with experts is essential for regularly updating the Web-based tool’s content and successful dissemination of the intervention broadly.

We demonstrate the feasibility of gaining consensus from health professionals through our own experiences during the development of a Web-based information tool. Prior to the consensus meeting, convened in relation to a Web-based tool for hematological patients and their support persons, we allocated sections of content to members for review based on their areas of expertise. Using a rating scale, the quality of the content was scored out of ten in areas such as accuracy, completeness, level of detail, and communication style, allowing identification of areas where improvements were needed. The feedback was then collated and presented at the advisory group meeting. There are various well-established approaches to obtaining consensus, such as the Delphi method, which may be used to determine the content of the Web program [68].

The consensus meeting provided a valuable opportunity to openly discuss the sections of information that were considered to require significant revisions by reviewers or topics where conflicting feedback was provided. To ensure such meetings are productive and efficient, conflict resolution techniques, such as voting, should be employed when consensus cannot be reached. When consensus cannot be reached and there is high level evidence (for example, clinical practice guidelines or evidence from Cochrane reviews) providing support for one particular intervention over another, the result should be in favor of the evidence. Where such evidence is not available, or the evidence is conflicting, all options should be presented for users. A transparent approach should be employed for communicating information where there is poor or conflicting evidence and no consensus among clinicians. An approach similar to that recommended by Raine and colleagues [69] may be used for increasing the transparency of recommendations where there may be conflicting evidence. Such an approach involves making explicit the reasons for disagreement and the degree of consensus, to assist with decision making.

Other advantages to involving multidisciplinary health care providers and researchers in a consensus meeting during the development phase of the Web-based tool include the opportunity to (1) discuss the acceptability of the intervention from a health care provider perspective, (2) investigate the probability of health care providers endorsing the information tool and promoting it to patients, and (3) strengthen relationships with clinical colleagues and foster potential future research collaboration.

#### Ensure a Well-Constructed and User-Friendly Interface

To increase the likelihood of use and effectiveness of a Web-based information tool, it is essential that the tool allow the user to extract the desired information as easily as possible. Most literature on effective Web page design emphasizes at least some of Dieter Ram’s design principles [70], namely that good design is innovative, useful, aesthetic, easily understood, unobtrusive, honest, durable, thorough, concerned with the environment, and has “as little design as possible”. Achievement of innovation without being obtrusive or over designed is interesting in the Web context: the current Web 2 browsers support user-provided content and hence user interaction; Web 3 (the *semantic* Web) adds contextual personalization; and the proposed Web 4 (the *symbiotic* Web) will be highly intelligent and fully executing. Each new Web form brings with it increased portability, pervasiveness, interactivity, and better support for multimedia (eg, video, audio) [71]. It is important that the Web-based tool uses innovation as appropriate to enhance communication, avoiding unaesthetic, obtrusive glitz by embracing elegant simplicity.

Another consideration is observation of the patterns followed by users’ eyes when reading Web pages [72]. Research shows that users typically read Web pages in an F-shaped pattern involving a pair of horizontal scans, the first across the top of the content, the second being lower and shorter, followed by a left-oriented vertical scan [72]. This has implications for Web page design as most users will not read all of a page’s content. The first lines of a page should state the most important information in the page, and headings, subheadings, bullet points, and paragraphs should start with words that impart information because these represent the left-most content.

With regards to the content imparted by the Web-based information tool, its design and implementation must be logical and intuitive to support information seeking for diverse users, including those who are less technologically savvy. The Web pages should be structured in a way that is meaningful to the user and easy to use [73]. Compliance with standard Web design conventions, such as positioning the navigation bar at the top and the organization’s logo in the top-left corner, allows users to easily understand the structure of the website based on their previous experience with other websites [73]. Incorporating various navigation aids, such as a search function, hyperlinks, tabbed menus, and sitemap, improves the ability of users to access desired information and offers navigation flexibility for browsing content [73,74]. Simplicity and consistency in design across all Web pages included in the intervention is essential for effective website navigation [73,74].

While content must be current, informative, and accurate, the visual design of the Web-based tool is important for capturing the user’s attention. Careful inclusion of graphic features, such as colors, images, and icons, helps to highlight key points and serve to increase the comprehensibility of text-based information [73]. Visual design features such as white space, contrast, and typography are also important considerations for maximizing readability. Ensuring the user interface is attractive and easy to navigate is likely to improve consumers’ use of and satisfaction with the Web-based information tool.

### Phase 2: Testing Early Iterations

#### Conduct Alpha and Beta Testing of Early Versions of the Tool

As early iterations of the Web-based tool are developed, it is essential to follow a test strategy, including alpha and beta testing, to assess the functionality of the tool and ensure it meets the objectives of the project. Alpha testing is often carried out within the project team and typically involves checking for issues such as incorrect or broken links, misspelled words, and problems loading multimedia objects [75]. Following this, beta testing may be undertaken, where the tool is tested by a sample of the intended end-users for any additional defects [75]. At this stage, initial feedback regarding usability may also be sought. During the preliminary testing phase, it may also be beneficial to conduct a heuristic evaluation of the user interface of the Web-based tool leading to improved usability.

#### Evaluate the Quality of the Tool Against Established Guidelines

Checklists and guidelines for assessing the quality of health-related information on the Internet have been developed; however, many are not supported by empirical evidence [76,77]. Several quality criteria for assessing information for consumers of health services include those developed by the King’s Fund, the United Kingdom National Health Service, and Coulter, Entwistle, and Gilbert [77]. These criteria emphasize the need for comprehensive and unbiased information that is presented in a way that is simple and easy to understand and can be integrated into clinical practice. Entwistle and colleagues advocate for additional dimensions of quality including relevance, accuracy, accessibility, comprehensibility, usability, and equity [78]. Other popular assessment tools include the DISCERN instrument [79] and IPDAS [49]. No gold standard quality criterion exists for assessing consumer health information, particularly when the intervention utilizes Internet technology. Evaluating a Web-based information tool against several criteria may provide the best measure of quality and serve to highlight areas where improvement is required.

#### Pilot the Web-Based Tool With Patients, Clinicians, and Other Stakeholders

Obtaining feedback from patients, their families, and other key stakeholders regarding the usability and acceptability of the tool is crucial for maximizing the probability of use and adoption in cancer care settings. Stakeholders are those who have a vested interest in the outcome of the project or initiative and are able to influence the direction it takes [80]. Prior to evaluating the effectiveness of the Web-based information tool, the tool should be pilot-tested with intended users. This procedure allows valuable consumer feedback to be obtained where participants can reflect on their own experiences and offer suggestions about what would have been most helpful to them and their families. The piloting process also provides an opportunity to examine whether the intervention is able to improve health literacy. Feedback from stakeholders could be collected through a variety of sources including surveys, qualitative interviews, or focus groups. Feedback obtained from stakeholders should be evaluated and incorporated prior to assessing the intervention’s effectiveness and included if recommended changes reflect the views of most consumers. Health care providers should again be consulted during this process to ensure the accuracy of amended information.

The involvement of clinicians throughout the previous development phase is advantageous in helping to ensure the Web-based information tool is acceptable to health care providers and readily integrated with current practice. It is, however, essential to conduct rigorous pilot testing of the acceptability and feasibility of the tool with clinicians who were not involved in Phase 1, given the role played by clinicians in tailoring the content of the tool. Similar techniques may be employed as for pilot testing with patients, such as surveys, qualitative interviews, or focus groups. Measurement of key strokes and eye movements can also be used to ensure that time demands are minimized and ease of use is maximized. This step is crucial for maximizing the probability that the tool will be integrated into clinical practice and adopted by clinicians in Phase 4.

### Phase 3: Testing for Effectiveness

#### Test the Effectiveness of the Web-Based Tool in Controlled Studies

Before the Web-based information tool can be disseminated and adopted, the uptake and effectiveness of the tool for improving patient outcomes should be evaluated with an adequate sample of consumers. Key issues central to the evaluation of effectiveness include: Does the target sample access the intervention? Which patient outcomes are likely to be impacted by the Web-based information tool? and How can effectiveness best be measured? The CONSORT-EHEALTH guidelines should be considered when designing and reporting studies examining the effectiveness of Web-based interventions [81]. While the involvement of consumers in development, as well as rigorous pilot testing conducted in previous phases, is likely to improve the likelihood that the target sample will utilize the Web-based information tool, controlled testing may reveal that uptake is influenced by factors that were not previously considered. It may be necessary to revisit previous phases in order to ascertain the barriers to uptake and revise the tool accordingly. If the intervention is found to be effective in improving patient outcomes, then steps can be taken to broadly disseminate the information tool in a range of clinical settings. Optimal intervention delivery and uptake relies on effective integration into clinical practice. It is essential that health care providers find the information tool acceptable in order to ensure adoption into practice. Partnerships with patient support organizations will help to ensure that the information tool is viewed as credible by providers.

### Phase 4: Integration and Implementation

#### Integrate the Tool Into Clinical Practice

Integration of a Web-based information tool is supported by its capacity to operate independently of health care provider and resource constraints. By automating and standardizing the provision of information, this approach minimizes the burden on physicians, reduces staffing costs, and increases patient convenience. While the burden on clinical staff is minimal, it is essential to provide education and training to ensure the innovative functions of the intervention are utilized to their potential. Educating clinical staff on the advantages of the information tool is likely to improve health care providers’ endorsement and encourage patient uptake. Training clinical staff to tailor the tool’s content to suit patients allows staff to maintain an active role in information provision without the added demands of traditional approaches.

Ensuring optimal intervention delivery and uptake is a complex issue faced by translational researchers and policy makers globally. The responsibility of educating and training clinical staff in the long term may be best managed by the medical college responsible for delivering care to the particular patient group. The application of evidence from Cochrane reviews [82-84] regarding effective methods of implementation into clinical practice is likely to be of significant benefit; however, this should be considered in conjunction with local protocols for health care delivery and information provision, which may differ between health care settings.

#### Monitor and Update the Web-Based Tool as New Evidence Becomes Available

Coulter, Entwistle, and Gilbert recommend that patient information be based on the best available evidence and be periodically reviewed and updated to reflect advancements [85]. Ensuring information is accurate and up-to-date is essential to informing patients of newly available treatment options and improved services. Partnership with patient support organizations may be extremely beneficial for assisting with this process, as they are likely to be in touch with the needs and preferences of consumers and aware of changing evidence within the field. Developers should also capitalize on the ability to track and record how the Web-based tool is used and obtain real-time feedback from consumers. This valuable data should be used as a means for evaluating and improving the Web-based tool regularly to address the information needs of consumers.

### Conclusions

New generation Web-based tools that are tailored and integrated into clinical care have the potential to overcome many of the limitations of general Web-based information, thus providing realizable benefits to patients and support persons. The proposed 4-phase DoTTI framework provides a model for a comprehensive approach to the development of Web-based informational tools for patients. The approach is evidence-informed, consumer-centered, flexible, and systematic. Implementation of the framework requires research at key phases including accessibility, acceptability, and effectiveness of each tool.

## Abbreviations

CD-ROM: compact disc, read-only-memory
CONSORT: Consolidated Standards of Reporting Trials
DoTTI: Design and develOpment, Testing early iterations, Testing for Effectiveness, Integration and implementation
DVD: digital versatile disc
IPDAS: International Patient Decision Aids Standards
